# Case report: A novel technique of robotic low-tension hiatal hernia repair using mediastinoplication

**DOI:** 10.1016/j.mex.2025.103376

**Published:** 2025-05-16

**Authors:** Carlos Eduardo Domene, Carlos Augusto Scussel Madalosso, Victor Ramos Mussa Dib, Paulo Reis Rizzo Esselin de Melo, André Valente Santana, William Carlos Giglio Mira Neto, Paula Volpe

**Affiliations:** a Integrated Center for Advanced Medicine (CIMAMED), 74 Dona Adma Jafet Street, Suite 162, Bela Vista, São Paulo - SP, 01308-050, Brazil; b Gastrobese Clinic, 1953 Uruguai St., 8th floor, 99010-111, Passo Fundo - RS, Brazil; c Victor Dib Institute, 1444 Álvaro Botelho Maia Ave, 69020-210, Manaus - AM, Brazil; d Paulo Reis Institute, Trade Center Building Condominium, 2nd Floor, Suite 202, 250 Street 10, Block B-6, Lot 5/9, Setor Oeste, Goiânia - GO, 74120-020, Brazil; e Alfredo Nasser University Center (UNIFAN), 26 Bela Vista Avenue, Jardim Esmeraldas, Goiânia - GO, 74905-020, Brazil; f Department of Surgery, Escola Paulista de Medicina, Federal University of São Paulo, 740 Botucatu Street, Vila Clementino, São Paulo - SP, 04023-062, Brazil; g Rede D´Or São Luiz Hospitals, 86 Rocio Street, Vila Olímpia, São Paulo - SP, 04552-050, Brazil; h Rede D´Or Hospital São Luiz Itaim, 95 Dr. Alceu de Campos Rodrigues Street, Vila Nova Conceição, São Paulo - SP, 04544-000, Brazil

**Keywords:** Robotic surgery, Mediastinoplication, Low-tension repair, Innovative surgical technique, Diaphragmatic crura, Abdominal-thoracic pressure gradient, Mediastinal dissection, Minimally invasive procedures, Giant hiatal hernia, Recurrent hiatal hernia, Robotic Low-Tension Hiatal Hernia Repair Using Mediastinoplication

## Abstract

Surgery for giant hiatal hernias presents significant challenges, with laparoscopic surgery widely regarded as the gold standard. This approach typically involves complete resection of the hernia sac, thorough esophageal mobilization, tension-free closure of the diaphragmatic crura, and proper fundoplication. Despite various strategies to reduce recurrence, such as mesh reinforcement, there is no consensus on their superiority over traditional methods. Robotic surgery introduces greater precision, particularly in complex cases involving large hernias (grades III and IV). It facilitates safer dissections and more effective esophageal mobilization, challenging the concept of a short esophagus. A novel technique, mediastinoplication, addresses the positive abdominal-thoracic pressure gradient, a key factor in hernia recurrence. By reducing mediastinal dead space and approximating mediastinal structures, this technique aims to minimize tension on the crura and reduce recurrence, seroma, hematoma, and abscess. While further validation of mediastinoplication's long-term efficacy is needed, it adheres to fundamental surgical principles and offers a promising solution to high recurrence rates. Future studies are essential to establish its role in standard practice.

• Introduces robotic mediastinoplication as a novel technique.
• Robotic suturing increases the feasibility of suturing mediastinal structures that are inaccessible by traditional laparoscopic methods.
• Mediastinoplication reduces tension, allowing a low-tension hiatal repair.

## Graphical abstract

**Figure:**
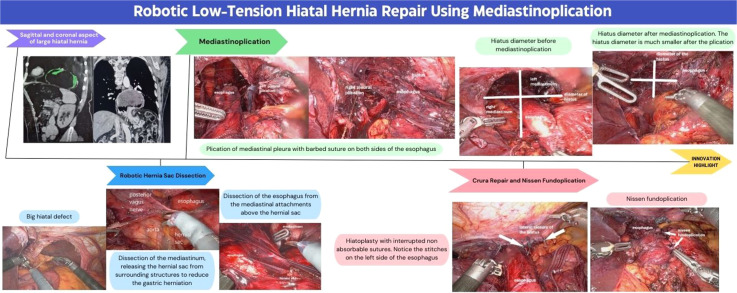

Specifications table

**Table:** 

Subject area:	Medicine and Dentistry
More specific subject area:	Robotic-assisted surgical techniques for the repair of giant hiatal hernias with mediastinal involvement.
Name of your method:	Robotic Low-Tension Hiatal Hernia Repair Using Mediastinoplication
Name and reference of original method:	Not applicable
Resource availability:	Robotic access

## Background

Hiatal hernia repairs are commonly performed to treat symptomatic gastroesophageal reflux disease (GERD) and prevent recurrence. The standard procedure typically involves hernia sac dissection, crura repair, gastropexy and Nissen fundoplication [1]. However, in cases of giant hiatal hernias, recurrence is frequent [2] with significant mediastinal involvement, achieving a tension-free repair can be challenging [3] which leads to many different inconclusive interventions such as use of absorbable or non-absorbable mesh, relaxing incisions [4] use of ligament teres [5] or a left triangular ligament flap [6]. Here, we present a novel approach utilizing mediastinoplication as part of a robotic-assisted low-tension hiatal hernia repair, which we found effective in ensuring a durable and tension-free repair.

## Method details

### Patient presentation

**Fig. 1:**
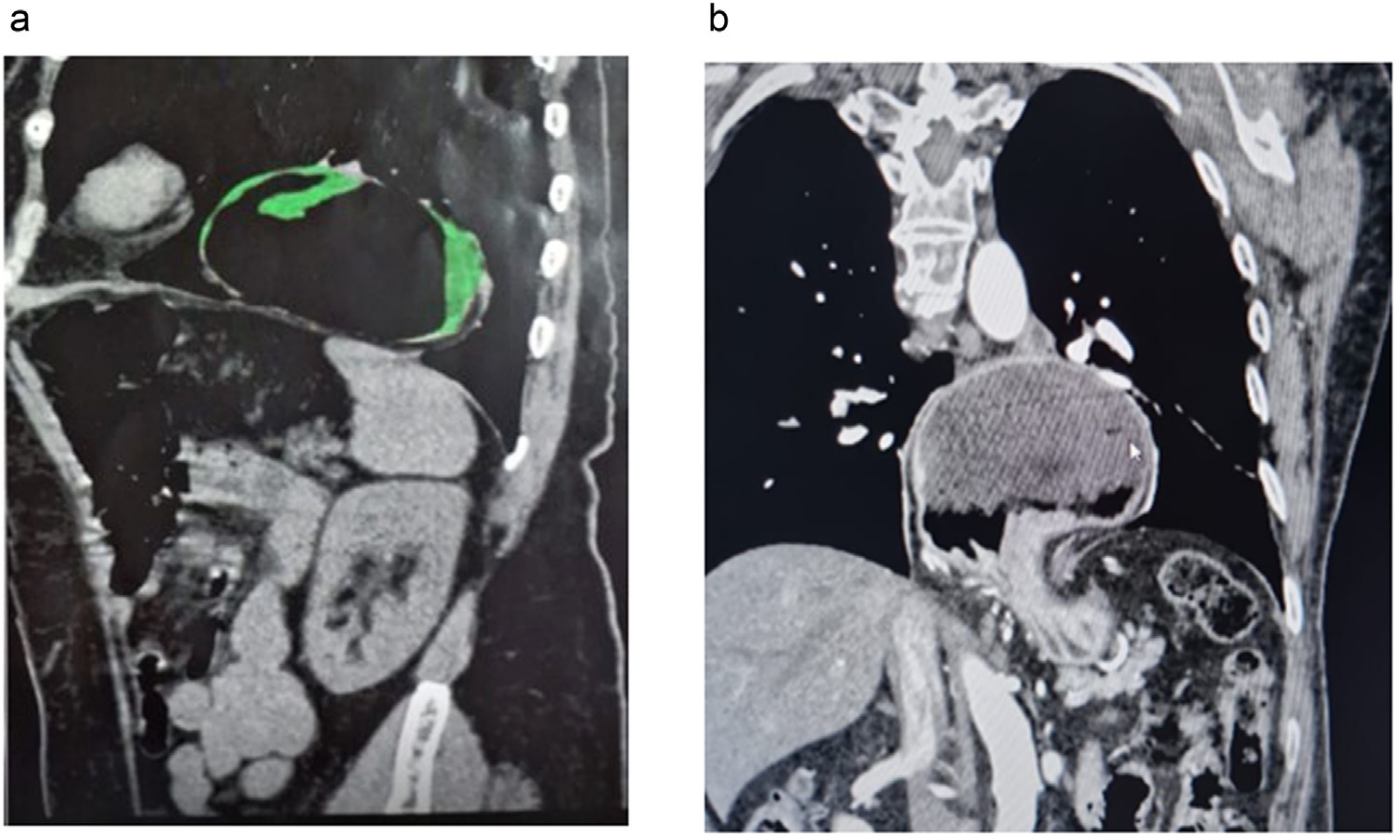
CT scan (right) showing sagittal and coronal aspect of large hiatal hernia with significant intrathoracic stomach and esophageal displacement.

A 65-year-old female, BMI 26.2Kg/m2, retired, on progesterone therapy, using PPI on demand, with a history of severe GERD presented with symptoms of dysphagia, regurgitation, and epigastric discomfort. Imaging studies, including an upper endoscopy, upper GI series and CT scan, revealed a large hiatal hernia with significant intrathoracic stomach and esophageal displacement – Fig. 1. No other comorbidities were referred. The patient was referred to surgical intervention.

### Surgical technique

#### Robotic hernia sac dissection

**Fig. 2:**
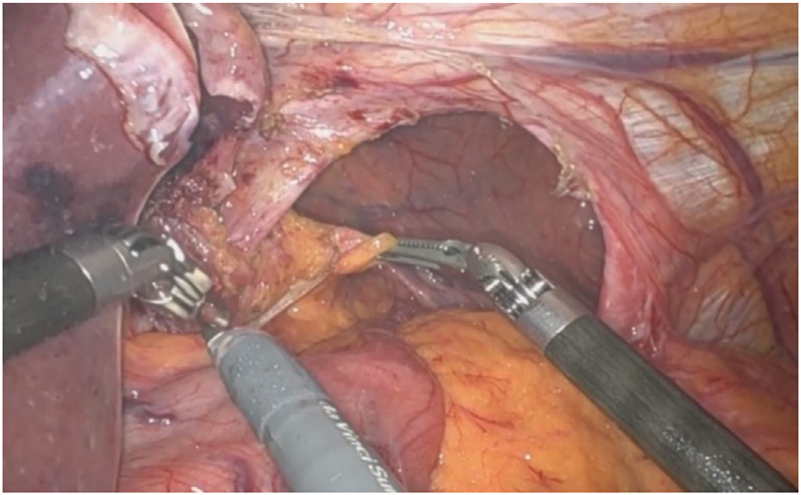
A big hiatal defect, with migration of almost all the stomach to the mediastinum.

**Fig. 3:**
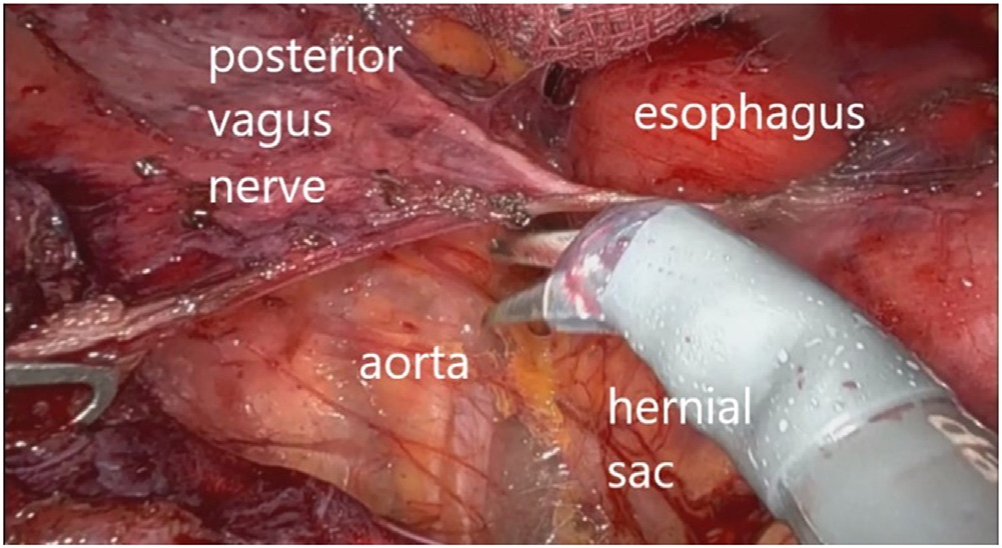
Dissection of the mediastinum, releasing the hernial sac from surrounding structures to reduce the gastric herniation.

**Fig. 4:**
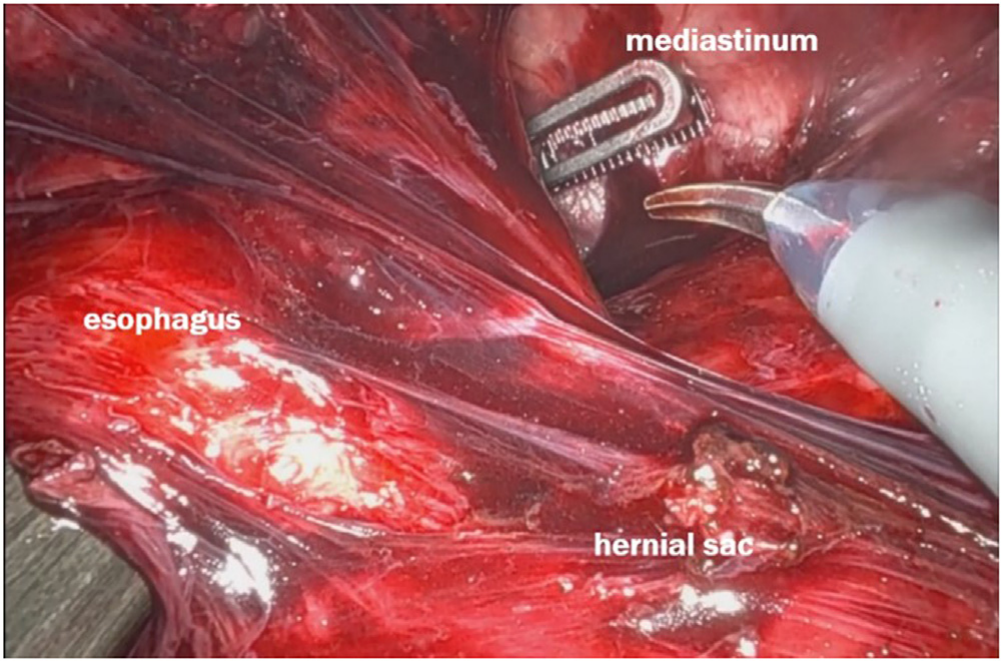
Dissection of the esophagus from the mediastinal attachments above the hernial sac.

The patient was positioned supine and placed under general anesthesia. Using a robotic-assisted approach, trocars were inserted after the abdomen was insufflated. There was a big hiatal defect, with migration of almost all the stomach to the mediastinum – Fig 2. A complete dissection of the hernia sac was performed, with careful attention to avoid injury to surrounding structures, including the vagus nerves – Fig 3. The hernia sac was fully mobilized from the mediastinum, allowing for clear exposure of the crura and esophagus [7,8] – Fig 4.

#### Esophageal mobilization

**Fig. 5:**
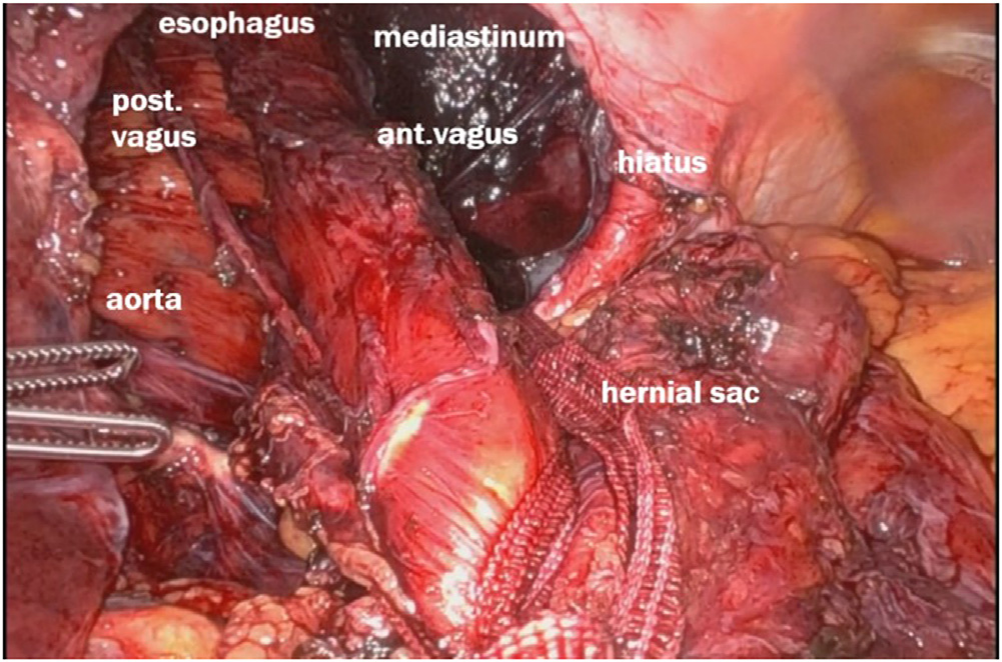
Esophagus fully mobilized from mediastinum, with good abdominal length.

The next step involved a complete mobilization of the esophagus within the mediastinum [9]. The esophagus was carefully dissected free from its mediastinal attachments, ensuring adequate length for a tension-free repair. This step was critical for preventing postoperative recurrence and ensuring that the esophagus could lie comfortably within the abdominal cavity even before a crura repair had been performed – Fig 5.

#### Mediastinoplication

**Fig. 6:**
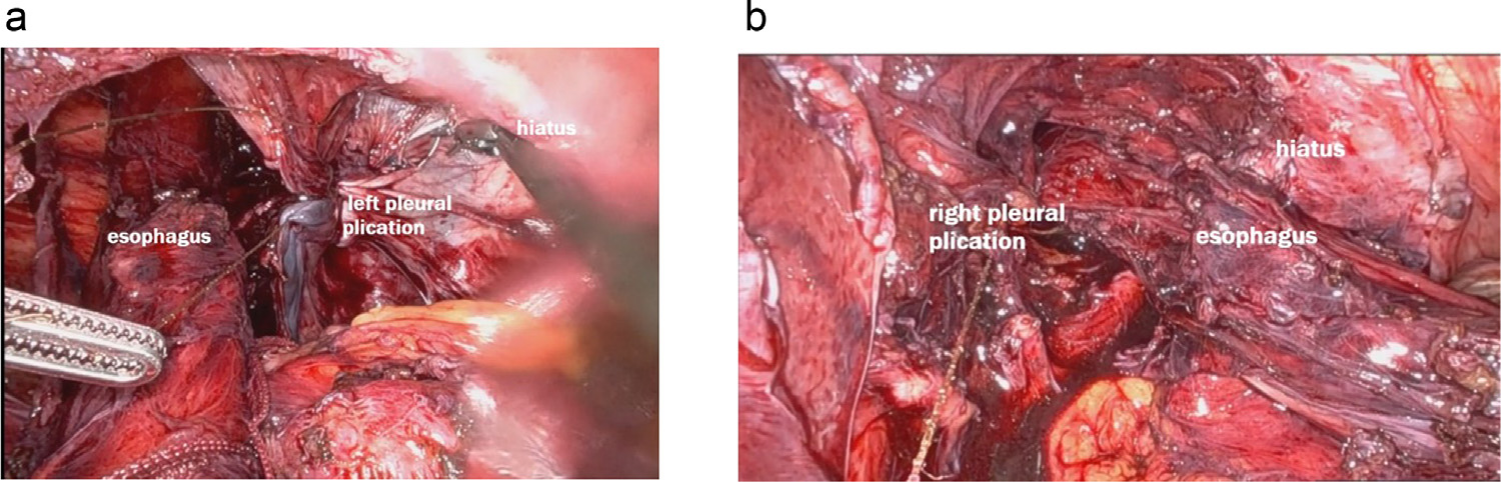
Plication of mediastinal pleura with barbed suture on both sides of the esophagus.

**Fig. 7:**
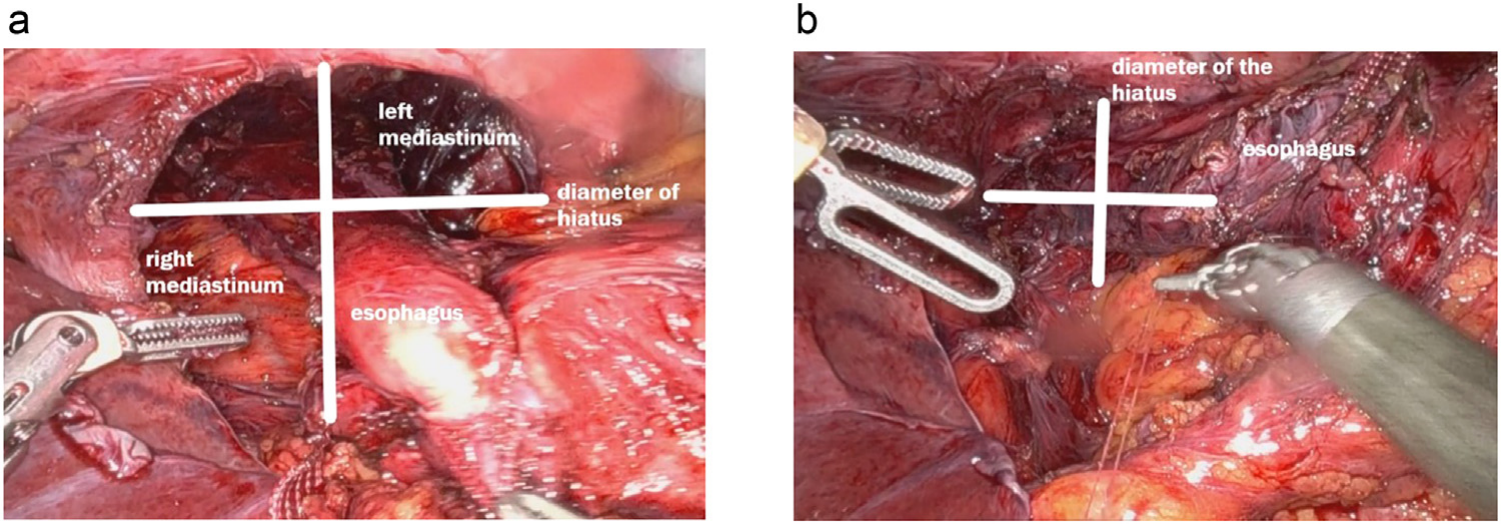
LEFT: Hiatus diameter before mediastinoplication. RIGHT: hiatus diameter after mediastinoplication. The hiatus diameter is much smaller after the plication.

A novel aspect of this procedure involved the plication of the mediastinal fibers, mediastinal pleura, and pericardium on both the right and left sides with barbed sutures [10] – Fig 6. This plication served to reduce the mediastinal space and tension. This prevents the esophagus from migrating back into the thoracic cavity. Separate sutures were placed on each side to obliterate the wide space remaining after hernia reduction. The plication effectively reduced the hollow volume of the mediastinum, creating a tighter and firm environment to keep the esophagus in place – Fig 7.

#### Crura repair

**Fig. 8:**
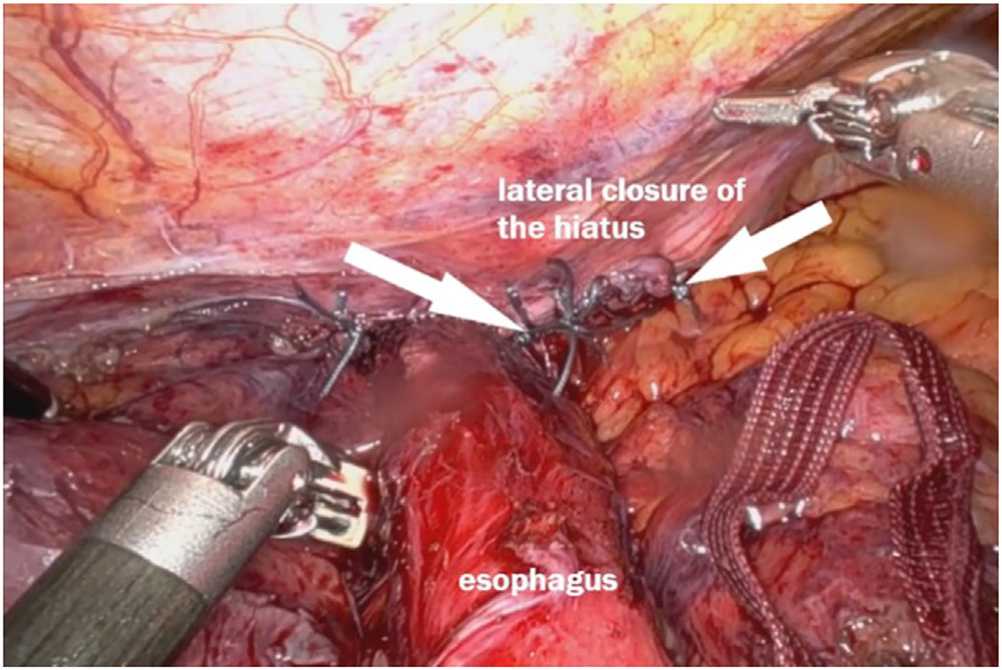
Hiatoplasty with interrupted non absorbable sutures. Notice the stitches on the left side of the esophagus.

Following the mediastinoplication, a reduced tension was also observed in the crura. This allowed a natural approximation of the right and the left crura. A tension-free repair was done by using non-absorbable sutures. A sequential testing of bougie progression through the hiatus was done to ensure that the esophagus could pass freely without constriction or obstacle. This step was repeated until a satisfactory hiatus closure was achieved, with no tension and adequate space for the esophagus – Fig 8.

#### Nissen fundoplication

**Fig. 9:**
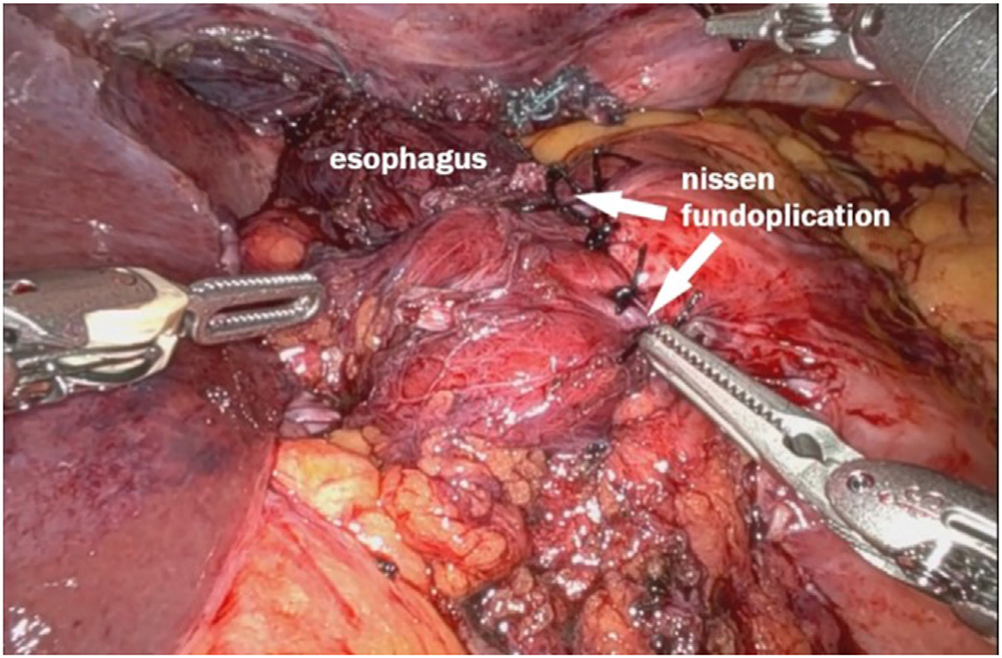
Nissen fundoplication.

Finally, a Nissen fundoplication was performed using the mobilized abdominal esophagus. The stomach was wrapped 360 degrees around the esophagus to create a valve mechanism that would prevent reflux. Given the adequate esophageal mobilization and the reduction in mediastinal space achieved through the mediastinoplication, the fundoplication was completed without tension. The lack of tension is crucial in preventing postoperative dysphagia or recurrence ensuring long-term success – Fig 9.

#### Operative time

The total operating time lasted 3 h and 48 min, and did not have any complication.

This video (link1) demonstrates the Robotic Low-Tension Hiatal Hernia Repair Using Mediastinoplication.

## Method validation

### Postoperative course

The patient tolerated the procedure well and was extubated in the operating room. Postoperatively, the patient was started on a liquid diet and advanced to soft solids over the course of a week. She was discharged on postoperative day 2 with minimal discomfort. At the 6-week follow-up, the patient reported a complete resolution of her preoperative symptoms, including dysphagia and regurgitation. A follow-up upper GI series showed no evidence of esophageal obstruction. Upper endoscopy did not find abnormalities with a hiatus properly calibrated.

## Limitations

We present a technique aimed at treating large and complex hiatal hernias characterized by an enlarged hiatus. This approach seeks to provide a safe, tension-free repair, eliminating the need for prostheses such as synthetic meshes. However, it is important to highlight certain limitations associated with the procedure, such as the requirement for robotic platforms to ensure safety during transhiatal mediastinal suturing, as well as adequate training in robotic surgery. Additionally, the results of this method must be monitored over time to identify potential recurrences or complications.

## Ethics statements

The authors confirm that informed consent was obtained from the patient involved in the study. This consent included permission to use clinical data and images for research and publication purposes, ensuring compliance with ethical standards and patient confidentiality.

## Related research article

None

## CRediT authorship contribution statement

**Carlos Eduardo Domene:** Conceptualization, Methodology, Investigation, Resources, Writing – original draft, Visualization. **Carlos Augusto Scussel Madalosso:** Resources, Writing – original draft, Visualization. **Victor Ramos Mussa Dib:** Writing – review & editing. **Paulo Reis Rizzo Esselin de Melo:** Writing – review & editing. **André Valente Santana:** Writing – review & editing. **William Carlos Giglio Mira Neto:** Writing – review & editing. **Paula Volpe:** Supervision, Writing – review & editing.

## Declaration of competing interest

The authors declare that they have no known competing financial interests or personal relationships that could have appeared to influence the work reported in this paper.

## Data Availability

No data was used for the research described in the article.

## Appendix Supplementary materials

Image, video 1
